# Understanding of prognosis in non-metastatic prostate cancer: a randomised comparative study of clinician estimates measured against the PREDICT *prostate* prognostic model

**DOI:** 10.1038/s41416-019-0569-4

**Published:** 2019-09-16

**Authors:** David R. Thurtle, Valerie Jenkins, Paul D. Pharoah, Vincent J. Gnanapragasam

**Affiliations:** 1 Academic Urology Group, University of Cambridge, Cambridge, UK; 2 Sussex Health Outcomes Research & Education in Cancer, University of Sussex, Brighton, UK; 3 Centre for Cancer Genetic Epidemiology, University of Cambridge, Cambridge, UK

**Keywords:** Prostate cancer, Surgical oncology, Nomograms

## Abstract

PREDICT *Prostate* is an individualised prognostic model that provides long-term survival estimates for men diagnosed with non-metastatic prostate cancer (www.prostate.predict.nhs.uk). In this study clinician estimates of survival were compared against model predictions and its potential value as a clinical tool was assessed. Prostate cancer (PCa) specialists were invited to participate in the study. 190 clinicians (63% urologists, 17% oncologists, 20% other) were randomised into two groups and shown 12 clinical vignettes through an online portal. Each group viewed opposing vignettes with clinical information alone, or alongside PREDICT *Prostate* estimates. 15-year clinician survival estimates were compared against model predictions and reported treatment recommendations with and without seeing PREDICT estimates were compared. 155 respondents (81.6%) reported counselling new PCa patients at least weekly. Clinician estimates of PCa-specific mortality exceeded PREDICT estimates in 10/12 vignettes. Their estimates for treatment survival benefit at 15 years were over-optimistic in every vignette, with mean clinician estimates more than 5-fold higher than PREDICT *Prostate* estimates. Concomitantly seeing PREDICT *Prostate* estimates led to significantly lower reported likelihoods of recommending radical treatment in 7/12 (58%) vignettes, particularly in older patients. These data suggest clinicians overestimate cancer-related mortality and radical treatment benefit. Using an individualised prognostic tool may help reduce overtreatment.

## Background

Decision-making around treatment for non-metastatic prostate cancer (PCa) is notoriously complex. Shared decision-making depends upon both clinician and patient having a good understanding of the benefits and harms of different management options. Estimation of life expectancy is known to be poor among PCa specialists.^1,2^ However, it is currently unknown how well clinicians estimate mortality risk from prostate cancer, nor how these perceptions affect treatment recommendations.

PREDICT *Prostate* is a new individualised prognostic model and decision aid developed and validated within cohorts of over 12,000 PCa patients.^3^ The model provides unbiased and personalised 15-year cancer-specific and overall survival estimates, alongside estimates of survival benefit from radical therapy compared with conservative management. Its accuracy is demonstrated by concordance indices in the region of 0.84 and the model has recently been successfully retested in an external validation cohort of over 69,000.^4^ PREDICT *Prostate* (www.prostate.predict.nhs.uk) can thus inform decisions with a quantifiable reference for prognosis. It has also recently been endorsed by the National Institute for Health and Care Excellence (NICE). In this study, we used this model as a standardised reference to compare against clinician estimates of prognosis and assessed its potential impact on treatment recommendations.

## Methods

**Fig. 1:**
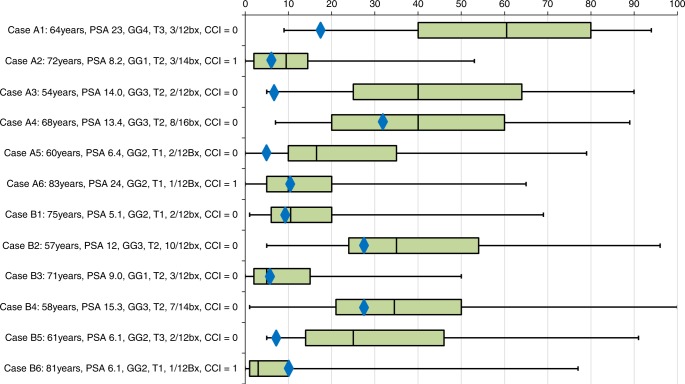
Boxplots showing the median, IQR and range of clinician estimated percentages of men dying of prostate cancer by 15 years after diagnosis without radical treatment for each of 12 case vignettes. For comparison, the PREDICT *Prostate* estimates for prostate cancer death by 15 years are shown by blue diamonds. PSA  prostate-specific antigen, T clinical tumour stage, GG grade group, bx biopsy cores, CCI Charlson Comorbidity Index

A randomised online virtual clinic was developed using Qualtrics® research software (Utah, USA). Prostate cancer specialists were invited to participate predominantly through professional mailing lists between June and September 2018. Respondents were encouraged to share the survey link with local colleagues. No incentive was offered for participation. Participants were randomised into two groups and shown 12 clinical vignettes of men with PCa: first 6 vignettes with clinical information alone, and then 6 vignettes with clinical information alongside PREDICT *Prostate* survival estimates. Each group saw the opposite 6 vignettes with and without the PREDICT estimates. Survey progression was uni-directional—preventing respondents from amending previous answers. Clinician estimates of 15-year survival outcomes were compared to PREDICT *Prostate* estimates. The likelihoods of recommending treatment were compared between the randomisation groups. Each case vignette was designed to represent scenarios in which use of the PREDICT *Prostate* tool might be appropriate (Fig. 1). The full questionnaires are available in Supplementary Information 1. Data analyses were performed using Stata 14 (Texas, USA). Responses were anonymised.

## Results

190 responses were received from 121 urologists (64% [85 consultants, 36 trainees]), 32 oncologists (17% [29 consultants, 3 trainees), 25 PCa specialist nurses (13.2%) and 12 other professionals (6%); henceforth collectively referred to as ‘clinicians’. Sixty percent of respondents reported working in specialist cancer centres and 82% reported counselling men with PCa at least weekly. 81% and 19% of respondents reported working in a UK, or non-UK centre, respectively. Clinician estimates of 15-year PCSM varied significantly and exceeded PREDICT *Prostate* estimates in most cases (83%) (Fig. 1). Mean clinician estimates of PCSM across all 12 cases were 1.9-fold greater than PREDICT *Prostate* estimates. Perceptions of survival benefit from upfront radical treatment at 15 years were similarly much higher, with mean clinician estimates of survival benefit 5.4-fold greater than the matched PREDICT *Prostate* estimates (Supplementary Table 1).

Likelihood of recommending treatment using clinical information alone correlated with clinician estimates of PCSM and with traditional three-stratum risk stratification criteria (Supplementary Table 1). It was also strongly influenced by patient age, with treatment recommendations particularly high (>80%) in younger men (<65years) with any ‘high risk’ features.^5^

**Fig. 2:**
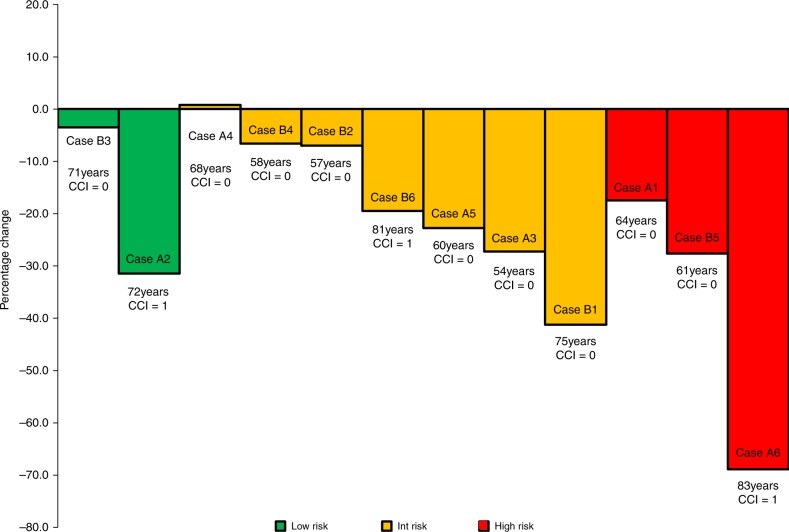
Mean difference in clinician likelihood of recommending radical treatment when shown PREDICT *Prostate* estimates in addition to routine diagnostic clinical information alone. Results for all 12 hypothetical cases are shown, sorted by EAU risk group. The case number, age and Charlson comorbidity index (CCI) is reported. Further case details are shown in Fig. 1 and Supplementary Information 1

Concomitantly viewing estimates from PREDICT *Prostate* led to reductions in likelihood of recommending radical treatment in 11/12 (92%) vignettes (Fig. 2), with significant differences (*p* < 0.05) in 7/12 (58%). Percentage decreases were most evident in intermediate risk cases, older patients (>70 years) and in the presence of comorbidity. For example, in a 75-year old man with PSA 5.1 and Gleason 3+4 disease in 2/12 biopsy cores (Case B1), the mean likelihood of recommending treatment fell from 32.5% with clinical information alone to 19.1% when PREDICT estimates were also shown (*p* = 0.009). Although reported likelihood of recommending treatment differed substantially between the two groups, the vast majority of respondents felt the PREDICT Prostate estimates were ‘similar to’ what they expected (Supplementary Fig. 1). Overall, 81% of respondents felt PREDICT *Prostate* would be a useful clinical tool.

## Discussion

Clinician understanding of PCa prognosis in non-metastatic disease is not well-explored in the literature. However, estimates of overall life expectancy in patients with PCa among urologists, oncologists and other health professionals are known to suffer from ‘inaccuracy, imprecision and inconsistency’.^1,2^ Our study suggests that PCa professionals generally overestimate cancer-related mortality. The results also suggest that clinicians’ perceptions of treatment effectiveness are generally over-optimistic; well in excess of a 75% improvement which does not correlate with direct evidence from RCTs.^6,7^

Our findings, that clinicians recommend radical treatment in most intermediate or high risk PCa, are unsurprising with the current reliance on a three-tier risk-stratification system. However, growing evidence supports the judicious use of surveillance in some men with intermediate-risk disease, and more individualised approaches are being sought.^8^ Overtreatment even of low-risk disease also remains a significant issue in PCa management, with up to 24% being managed with radical therapy in some centres in the UK.^9^ Our results suggest that by providing individualised and contextualised prognostic information, PREDICT *Prostate* may prompt clinicians to re-evaluate prognosis estimation and increase consideration of non-interventional strategies. This may be particularly true for older patients, or those with comorbidities, where the modelled overall survival benefits from treatment in PREDICT are adjusted for competing risks, rather than relying upon cancer-specific survival alone.

This study has many strengths as it represents the reported behaviour of a diverse spectrum of prostate cancer professionals within a randomised design and where the pre-defined sample size was exceeded. However, we recognise the study limitations inherent to questionnaire-based research. These findings were from a predominantly UK context and stated change in recommendations may not equal actual change in practice. The reported profession of respondents cannot be confirmed, and the final response rate cannot be determined due to the method of recruitment. Some respondents, may rarely manage patients with the localised prostate cancer characteristics described in the vignettes, however, all would be expected to partake in prostate cancer multi-disciplinary team (MDT) meetings. The survival endpoint of 15 years may also be unfamiliar, and we fully acknowledge that clinicians may recommend a therapy, but final treatment decisions are made by the patient himself. Nonetheless, physician recommendations do remain very important in guiding patients’ decisions.^10^ A randomised impact study of the PREDICT *Prostate* tool with patients is currently underway and a wider study to measure the actual change in treatment uptake is being planned.

In summary, this study suggests that PCa specialists appear to overestimate PCa-related mortality and the survival benefits of radical treatment for non-metastatic PCa. Using a freely available tool such as PREDICT *Prostate* can provide individualised and contextualised prognostic information which standardises the information patients receive. This may then help reduce variability in recommendations and facilitate unbiased and better-informed clinician-patient discussions. In turn, this may aid the reduction of overtreatment of good prognosis disease while also increasing the confidence that radical treatment, when needed, will confer a survival benefit and justify the risks of side effects. At the very basic level, it should enhance patient’s knowledge of risks and benefits and their ability to participate in their care.

## Supplementary information

Supplementary Files

## Data Availability

The datasets used and analysed during the current study are available from the corresponding author on reasonable request.

## References

[1] Walz J, Gallina A, Perrotte P, Jeldres C, Trinh QD, Hutterer GC (2007). Clinicians are poor raters of life-expectancy before radical prostatectomy or definitive radiotherapy for localized prostate cancer. BJu Int..

[2] Wilson JR, Clarke MG, Ewings P, Graham JD, MacDonagh R (2005). The assessment of patient life-expectancy: how accurate are urologists and oncologists?. BJU Int..

[3] Thurtle DR, Greenberg DC, Lee LS, Huang HH, Pharoah PD, Gnanapragasam VJ (2019). Individual prognosis at diagnosis in nonmetastatic prostate cancer: development and external validation of the PREDICT Prostate multivariable model. PLoS Med.

[4] Thurtle D., Bratt O., Stattin P., Pharoah P.D., Gnanapragasam V.J. (2019). External validation of the PREDICT Prostate tool for prognostication in non-metastatic prostate cancer: A study in 69,206 men from prostate cancer data base Sweden. European Urology Supplements.

[5] Heidenreich A, Bastian PJ, Bellmunt J, Bolla M, Joniau S, van der Kwast T (2014). EAU guidelines on prostate cancer. part 1: screening, diagnosis, and local treatment with curative intent-update 2013. Eur. Urol..

[6] Bill-Axelson A, Holmberg L, Garmo H, Rider JR, Taari K, Busch C (2014). Radical prostatectomy or watchful waiting in early prostate cancer. N. Engl. J. Med..

[7] Hamdy FC, Donovan JL, Lane JA, Mason M, Metcalfe C, Holding P (2016). 10-Year outcomes after monitoring, surgery, or radiotherapy for localized prostate cancer. N. Engl. J. Med..

[8] Dall’Era MA, Klotz L (2017). Active surveillance for intermediate-risk prostate cancer. Prostate Cancer Prostatic Dis..

[9] NPCA, National Prostate Cancer Audit - Annual Report 2017.

[10] Xu JP, Dailey RK, Eggly S, Neale AV, Schwartz KL (2011). Men’s perspectives on selecting their prostate cancer treatment. J. Natl Med. Assoc..

