# Supplementary material for: Understanding of prognosis in non-metastatic prostate cancer: a randomised comparative study of clinician estimates measured against the PREDICT prostate prognostic model
Source: Br J Cancer. 2019 Sep 16;121(8):715–8. doi: 10.1038/s41416-019-0569-4 (PMC6889281; doi:10.1038/s41416-019-0569-4)
Supplement: Supplementary file 1 — Supplementary Files [file 41416_2019_569_MOESM1_ESM.docx]

**Supplementary Files**

**Supplementary Information 1:** Research Survey (delivered through Qualtrics research software)

**Supplementary Table 1:** Clinician-reported mean prostate cancer mortality, non-prostate cancer mortality and overall survival benefit from treatment compared with conservative management. Clinician estimated percentages of men dying by 15 years without radical treatment for each of 12 case vignettes shown are compared with PREDICT Prostate estimates.

**Supplementary Figure 1:** The proportion of respondents that thought the PREDICT *Prostate* estimates for 15-year survival benefit from radical treatment were greater, less or similar to what they expected for each case vignette.

**Supplementary Information 1:** Research Survey (delivered through Qualtrics research software)

**Common starting Questions:**

1. What is your current role?
   1. Consultant urologist
   2. Consultant oncologist
   3. Trainee urologist
   4. Trainee oncologist
   5. Specialist Nurse
   6. Other (open box)
2. Which of the following best describes your primary place of work?
   1. UK tertiary cancer centre
   2. UK general hospital
   3. Non-UK specialist/academic centre
   4. Non-UK general hospital
   5. Other (open box)

IF – c or d … what is your country of work?

1. How often do you counsel men with newly diagnosed prostate cancer?
   1. Daily
   2. Weekly
   3. Monthly
   4. Rarely
   5. Never
2. When considering treatment options in newly diagnosed non-metastatic prostate cancer, in your routine clinical practice do you use nomograms or other risk prediction/stratification tools to help predict survival or aid decision making?
   1. Yes
   2. No

– If yes… which tool/nomogram(s) do you use?

1. When considering treatment as opposed to surveillance for prostate cancer, a prediction tool would be most useful when the risk of 10-year prostate cancer death is: 
   0-10%

10-20%

20-40%

>40%
All of the above

**RANDOMISATION**

**‘RANDOM GROUP 1’**

“A series of 6 brief clinical vignettes will be presented. 

From the available information for each case, please answer the questions below. ‘Radical treatment’ relates to prostatectomy OR radiotherapy. The questions are the same for each of these cases.”

1. Case B1

75 years old
PSA 5.1ng/ml
T1
Gleason 3+4 (grade group 2) in 2/12 biopsies.
No significant comorbidity.

1. On a scale from 0 (certainly not) to 100 (certainly) how likely would you be to recommend radical treatment? (0-100 slider)
2. Of 100 men with these characteristics, how many would you estimate will die from prostate cancer within 15 years if conservatively managed? (0-100)
3. Of 100 men with these characteristics, how many would you estimate will die from other causes (not prostate cancer) in 15 years? (0-100)
4. Of 100 men with these characteristics, if all were radically treated as opposed to conservatively managed, how many extra men would you estimate to be alive at 15 years? (0-100)
5. Case B2:

57 years old
PSA 12.0ng/ml
T2
Gleason 4+3 (grade group 3) prostate cancer in 10/12 cores.
No significant comorbidity.

1. Case B3
   71 years old
   PSA 9.0ng/ml
   T2
   Gleason 3+3  (grade group 1) disease in 3/12 cores.
   No significant comorbidity.
2. Case B4

58 years old
PSA 15.3 ng/ml
T2
Gleason 4+3  (grade group 3) disease in 7/14 cores.
No significant comorbidity.

1. Case B5

61 years old
PSA 6.1ng/ml
T3
Gleason 3+4  (grade group 2) in 2/12 cores.
No significant comorbidity.

1. Case B6

81 years old
PSA 6.1ng/ml
T1
Gleason 3+4  (grade group 2) in 1/12 cores.
Otherwise well, with no hospital admissions in the last 2 years.

The second group of clinical vignettes will be accompanied by some of the available output from PREDICT: Prostate. Please read the following vignettes and review the output then answer the questions below.

1. Case A1:

64 years old

PSA 23ng/ml

T3
Gleason 4+4 (grade group 4) in 3/12 biopsies.
Otherwise well with no hospital admissions in the last 2 years.

1. On a scale from 0 (certainly not) to 100 (certainly) how likely would you be to recommend radical treatment?
2. Considering the PREDICT estimates of survival benefit from radical treatment in this case (A1) :

The overall survival benefits of treatment are greater than I expected (button)

The overall survival benefits of treatment are less than I expected (button)
The overall survival benefits of treatment are similar to what I expected (button)

1. Case A2:

72 years old
PSA 8.2ng/ml.

T2
Gleason 3+3 (grade group 1) in 3/14 biopsy cores.
He has a history of myocardial infarction 1 year ago. 

1. Case A3:

54 years old
PSA 14.0ng/ml

T2
Gleason 4+3 (grade group 3) in 2/12 biopsies
Otherwise well with no hospital admissions in the last 2 years. 

1. Case A4:

68 years old
PSA 13.4ng/ml

T2 prostate
Gleason 4+3 (grade group 3) in 8/16 biopsies
Otherwise well with no hospital admissions in the last 2 years.

1. Case A5:

60 years old
PSA 6.4ng/ml
T1
Gleason 3+4 (grade group 2) in 2/12 biopsies.
Otherwise well with no hospital admissions in the last 2 years. 

1. Case A6:

83 years old
PSA 24ng/ml
T1
Gleason 3+4 (grade group 2) in 1/12 biopsies.
Admitted to hospital earlier this year with an ischaemic toe.

**‘RANDOM GROUP 2’**

1. Case A1:

64 years old

PSA 23.0 ng/ml

T3
Gleason 4+4 (grade group 4) in 3/12 biopsies.
Otherwise well with no hospital admissions in the last 2 years.

1. On a scale from 0 (certainly not) to 100 (certainly) how likely would you be to recommend radical treatment? (0-100 slider)
2. Of 100 men with these characteristics, how many would you estimate will die from prostate cancer within 15 years if conservatively managed? (0-100)
3. Of 100 men with these characteristics, how many would you estimate will die from other causes (not prostate cancer) in 15 years? (0-100)
4. Of 100 men with these characteristics, if all were radically treated as opposed to conservatively managed, how many extra men would you estimate to be alive at 15 years? (0-100)
5. Case A2:

72 years old
PSA 8.2ng/ml.

T2
Gleason 3+3 (grade group 1) in 3/14 biopsy cores.
He has a history of myocardial infarction 1 year ago. 

1. Case A3:

54 years old
PSA 14.0ng/ml

T2
Gleason 4+3 (grade group 3) in 2/12 biopsies
No significant comorbidity.

1. Case A4:

68 years old
PSA 13.4ng/ml

T2 prostate
Gleason 4+3 (grade group 3) in 8/16 biopsies
No significant comorbidity.

1. Case A5:

60 years old
PSA 6.4ng/ml
T1
Gleason 3+4 (grade group 2) in 2/12 biopsies.
No significant comorbidity.

1. Case A6:

83 years old
PSA 24.0 ng/ml
T1
Gleason 3+4 (grade group 2) in 1/12 biopsies.
Admitted to hospital earlier this year with an ischaemic toe.

The second group of clinical vignettes will be accompanied by some of the available output from PREDICT: Prostate. Please read the following vignettes and review the output then answer the questions below.

1. Case B1

75 years old
PSA 5.1ng/ml
T1
Gleason 3+4 (grade group 2) in 2/12 biopsies.
No significant comorbidity.

1. On a scale from 0 (certainly not) to 100 (certainly) how likely would you be to recommend radical treatment?
2. Considering the PREDICT estimates …

The overall survival benefits of treatment are greater than I expected (button)

The overall survival benefits of treatment are less than I expected (button)
The overall survival benefits of treatment are similar to what I expected (button)

1. Case B2:

57 years old
PSA 12.0ng/ml
T2
Gleason 4+3 (grade group 3) prostate cancer in 10/12 cores.
No significant comorbidity.

1. Case B3

71 years old
PSA 9.0ng/ml
T2
Gleason 3+3  (grade group 1) disease in 3/12 cores.
No significant comorbidity.

1. Case B4

58 years old
PSA 15.3 ng/ml
T2
Gleason 4+3  (grade group 3) disease in 7/14 cores.
No significant comorbidity.

1. Case B5

61 years old
PSA 6.1ng/ml
T3
Gleason 3+4  (grade group 2) in 2/12 cores.
No significant comorbidity.

1. Case B6

81 years old
PSA 6.1ng/ml
T1
Gleason 3+4  (grade group 2) in 1/12 cores.
No significant comorbidity.

**Common Closing Questions**

1. Do you feel PREDICT: *Prostate* would be a useful clinical tool?
   1. No
   2. Yes
   3. Unsure

31. Please enter any additional comments or feedback you have about PREDICT: *Prostate*  (Open box)

**Thank you for taking the time to complete this survey.**

|  | **Clinician estimates** | | | **PREDICT Prostate estimates** | | |
| --- | --- | --- | --- | --- | --- | --- |
| **Case Summary** | **15year PCa deaths** | **15year Non-PCa deaths** | **Extra men alive with radical treatment** | **15year PCa deaths** | **15year Non-PCa deaths** | **Extra men alive with radical treatment** |
|  | **Mean (95%CI)** | **Mean (95%CI)** | **Mean (95%CI)** |  |  |  |
| Case A1: 64yrs, PSA 23, GG4, T3, 3/12bx, Co=0 | 58.4 (91.5-98.7) | 31.4 (25.9-36.9) | 40.0 (33.9-46.0) | 17.8 | 20.6 | 7.2 |
| Case A2: 72yrs, PSA 8.2, GG1, T2, 3/14bx, Co=1 | 12.3 (8.9-15.7) | 62.8 (56.4-69.1) | 15.9 (9.9-21.9) | 8.0 | 67.6 | 1.2 |
| Case A3: 54yrs, PSA 14.0, GG3, T2, 2/12bx, Co=0 | 44.9 (38.5-51.2) | 22.6 (16.9-28.2) | 42.3 (35.0-49.5) | 9.2 | 7.1 | 4.3 |
| Case A4: 68yrs, PSA 13.4, GG3, T2, 8/16bx, Co=0 | 40.7 (34.8-46.6) | 40.9 (34.8-46.5) | 31.6(25.1-38.1) | 32.0 | 28.4 | 11.1 |
| Case A5: 60yrs, PSA 6.4, GG2, T1, 2/12Bx, Co=0 | 23.7 (18.4-29.1) | 29.9 (23.8-36.0) | 23.0 (16.3-29.8) | 6.2 | 14.1 | 2.7 |
| Case A6: 83yrs, PSA 24, GG2, T1, 1/12Bx Co=1 | 15.3 (11.0-19.5) | 82.0 (76.9-87.1) | 7.7 (4.6-10.8) | 11.8 | 87.8 | 0.1 |
| Case B1: 75yrs, PSA 5.1, GG2, T1, 2/12bx, Co=0 | 14.7 (11.8-17.6) | 61.7 (56.6-66.8) | 19.8 (14.1-25.5) | 9.4 | 57.9 | 1.8 |
| Case B2: 57yrs, PSA 12, GG3, T2, 10/12bx, Co=0 | 41.9 (35.7 - 48.1) | 18.4 (14.5-22.2) | 38.7 (31.4-46.0) | 27.2 | 9.7 | 11.9 |
| Case B3: 71yrs, PSA 9.0, GG1, T2, 3/12bx, Co=0 | 9.8 (6.8-12.8) | 50.1 (43.6-56.6) | 19.0 (11.5-26.6) | 8.0 | 42.7 | 2.3 |
| Case B4: 58yrs, PSA 15.3, GG3, T2, 7/14bx, Co=0 | 38.3 (32.0-44.6) | 21.4 (16.9-26.0) | 33.3 (26.6-40.4) | 28.6 | 10.7 | 12.3 |
| Case B5: 61yrs, PSA 6.1, GG2, T3, 2/12bx, Co=0 | 32.2 (25.9-38.6) | 26.2 (21.4-31.0) | 31.0 (23.4-38.6) | 9.1 | 15.5 | 3.9 |
| Case B6: 81yrs, PSA 6.1, GG2, T1, 1/12Bx Co=1 | 8.9 (4.9-12.9) | 79.6 (74.3-84.9) | 12.1 (5.7-18.5) | 10.3 | 88.1 | 0.1 |

**Supplementary Table 1**. Clinician-reported mean prostate cancer mortality, non-prostate cancer mortality and overall survival benefit from treatment compared with conservative management. Clinician estimated percentages of men dying by 15 years without radical treatment for each of 12 case vignettes shown are compared with PREDICT Prostate estimates. PSA = Prostate specific antigen; T = clinical tumour stage’ GG = grade group; Bx = biopsy cores; CCI = Charlson Comorbidity Index.


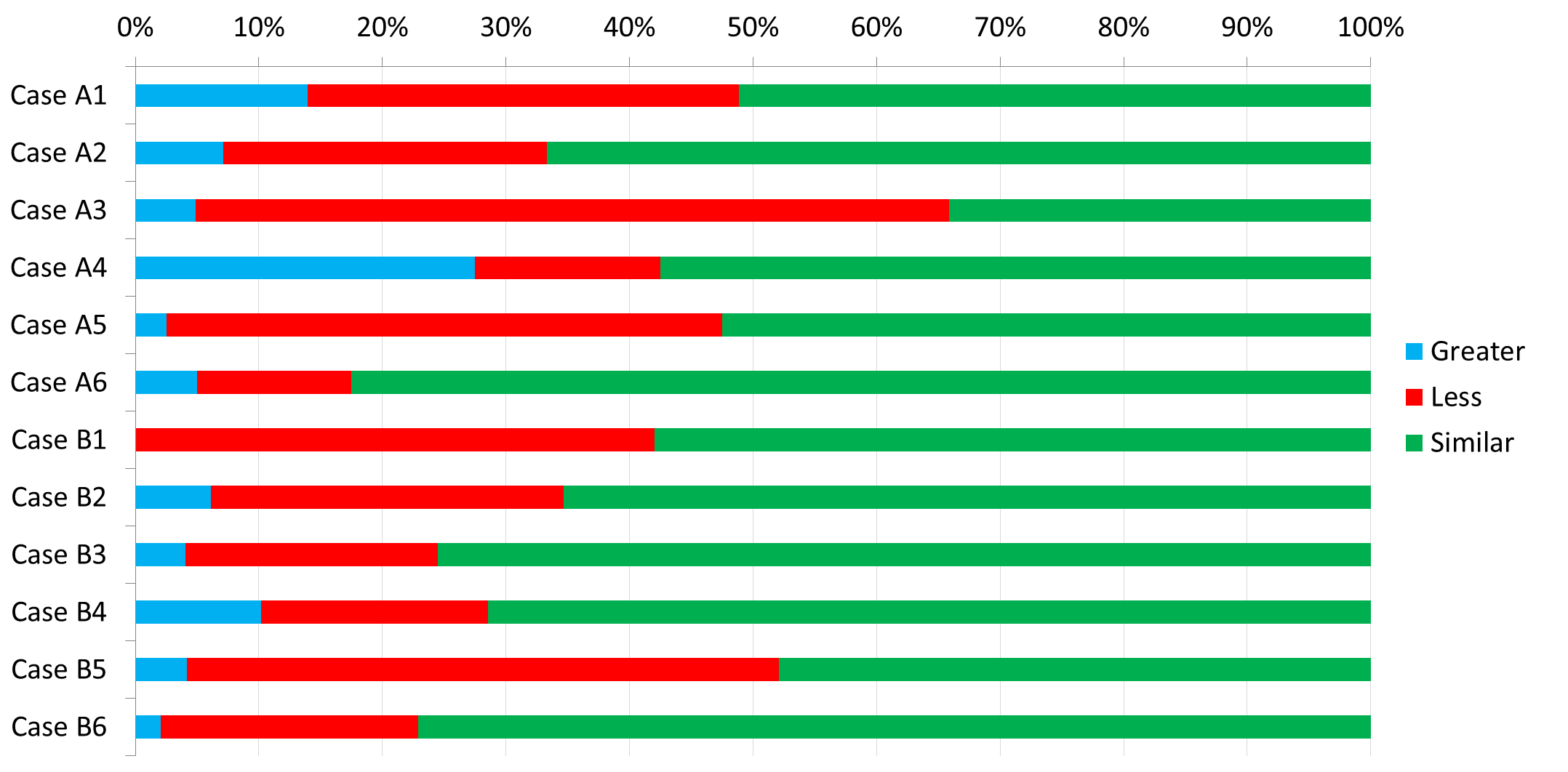


**Supplementary Figure 1** The proportion of respondents that thought the PREDICT *Prostate* estimates for 15-year survival benefit from radical treatment were greater, less or similar to what they expected for each case vignette.
